# Recent advances in perinatal neuroprotection

**DOI:** 10.12688/f1000research.20722.1

**Published:** 2019-11-29

**Authors:** Samata Singhi, Michael Johnston

**Affiliations:** 1 Department of Neurology, Kennedy Krieger Institute, Baltimore, Maryland, 21205, USA; 2 Department of Pediatric Neurology, Johns Hopkins Medicine, Baltimore, MD, 21287, USA

**Keywords:** perinatal, neuroprotection

## Abstract

Perinatal brain injury is a major cause of neurological disability in both premature and term infants. In this review, we summarize the evidence behind some established neuroprotective practices such as administration of antenatal steroids, intrapartum magnesium for preterm delivery, and therapeutic hypothermia. In addition, we examine emerging practices such as delayed cord clamping, postnatal magnesium administration, recombinant erythropoietin, and non-steroidal anti-inflammatory agents and finally inform the reader about novel interventions, some of which are currently in trials, such as xenon, melatonin, topiramate, allopurinol, creatine, and autologous cord cell therapy.

## Introduction

Perinatal brain injury is a major cause of neurological disability in both premature and term infants ^1^ and may include disorders of hearing, vision, speech, motor function, intellectual disability, and seizures. Therefore, preventive and restorative strategies for perinatal brain injury are critically needed to minimize adverse neurological sequelae. In this review, we discuss the established and emerging interventions for perinatal neuroprotection in term and preterm infants.

## Prevention of preterm delivery

Prematurity is the leading cause of morbidity and mortality in childhood within the developed world ^2^. Preterm birth (and low birth weight independently) is a leading risk factor for cerebral palsy (CP) and associated neurologic impairments and neurosensory disabilities ^3, 4^. Therefore, prevention of preterm delivery is a crucial strategy for perinatal neuroprotection.

## Antenatal steroids

A Cochrane systematic review including 30 studies (7774 women and 8158 infants) mostly from high-income countries found that treatment with antenatal corticosteroids (dexamethasone or betamethasone) as compared with placebo or no treatment is associated with a reduction in perinatal death (relative risk [RR] 0.72, 95% confidence interval [CI] 0.58 to 0.89), neonatal death (RR 0.69, 95% CI 0.59 to 0.81), and intraventricular hemorrhage (IVH) (RR 0.55, 95% CI 0.40 to 0.76) ^5^. Treatment with corticosteroids was associated with less developmental delay in childhood, although the data were deemed insufficient.

Antenatal steroids promote lung maturation ^6^, thereby stabilizing respiratory and hemodynamic system. In addition, they stabilize germinal matrix vasculature ^7, 8^ and exert vasoconstrictive effects on fetal cerebral blood flow, thereby offering protection against IVH and hypercapnia-induced vasodilatation ^9, 10^.

Antenatal corticosteroid administration in women at risk of preterm birth is the standard of care. However, further research is warranted to support this practice in lower-income settings and high-risk obstetric groups.

## Magnesium sulfate

Several randomized controlled trials (RCTs) have demonstrated the neuroprotective effects of antenatal magnesium sulfate in preterm infants ^11– 15^. A recent meta-analysis that included the above-mentioned trials concluded that antenatal magnesium sulfate given prior to preterm birth for fetal neuroprotection (4448 babies) prevents CP (mild, moderate, and severe) and reduces the combined risk of fetal/infant death or CP (RR 0.86, 95% CI 0.75 to 0.99) ^16^. This benefit was seen independently of reason for preterm birth with similar effects across a range of preterm gestational ages. (It should be noted that the trials included in this analysis included women at less than 33 weeks’ gestation.) These results were consistent with previous meta-analyses that found that magnesium sulfate administered to women at high risk of delivery before 34 weeks of gestation reduced the risk of CP and rate of gross motor dysfunction ^17– 19^. Antenatal magnesium sulfate is also associated with reduced cerebellar hemorrhage on magnetic resonance imaging (MRI) in preterm newborns ^20^. However, long-term follow-up has not demonstrated improved neurological, cognitive, behavioral, or functional outcomes in school age for children of women receiving magnesium sulfate for preterm delivery (<30 weeks) ^21, 22^.

Based on the above data, antenatal magnesium remains the standard of care for women at less than 32 weeks’ gestation who are at risk for imminent delivery. Evidence for effectiveness between 34 to 37 weeks remains to be established.

Recent studies have also demonstrated improvements in short-term neurological outcomes after postnatal magnesium sulfate infusion. Two small RCTs using postnatal magnesium sulfate infusion (250 mg/kg per day) for 3 days in term neonates with severe birth asphyxia resulted in an improved survival with normal results of cranial computed tomography and electroencephalography in the treated group compared with the control group ^23, 24^. However, no significant neurodevelopmental improvement was noted at 6 months ^25^. A prospective observational study, however, reported normal neurodevelopmental outcomes at 18 months in 73% of infants with moderate to severe hypoxic ischemic encephalopathy (HIE) treated with magnesium sulfate (in combination with dopamine) within 6 hours of birth ^26^. A multicenter RCT of therapeutic hypothermia plus magnesium sulfate versus hypothermia alone of term and near term newborn infants born at, at least 35 weeks (the Mag Cool Study) with a clinical diagnosis of moderate or severe HIE found no differences in the short-term adverse outcomes (death, seizures, and intracranial hemorrhage) between the two groups ^27^.

The mechanism underlying the neuroprotective effects of magnesium sulfate is not well elucidated. It is widely accepted that magnesium prevents excitotoxic damage through *N*-Methyl- d-aspartic acid (NMDA) receptor blockade ^28^. Moreover, magnesium has anti-inflammatory properties ^29^ and reduces the production of pro-inflammatory cytokines interleukin-6 and tumor necrosis factor-alpha ^30^. Animal models have also demonstrated that magnesium sulfate changes expression of several genes, thereby altering the mitochondrial and metabolic substrate of the immature brain and reducing vulnerability to hypoxia ^31^. Therefore, magnesium-induced preconditioning of the brain via development of mitochondrial resistance and suppression of inflammation likely contributes to its mechanism of perinatal protection ^32^.

As advances in neonatal care enable increased survival of infants of 22 to 23 weeks’ gestational age, studies will need to be carried out in this population to determine the effectiveness of interventions.

## Delayed umbilical cord clamping

Delayed cord clamping is typically defined as a lapse of at least 30 to 60 seconds before clamping the umbilical cord after delivery. In term infants, a meta-analysis of 15 trials involving a total of 3911 women and infant pairs found no significant differences between early (<60 seconds) and late (>60 seconds) clamping in terms of neonatal mortality (RR 0.37, 95% CI 0.04 to 3.41) or for most other neonatal morbidity outcomes ^33^. However, mean birth weight was significantly higher in the late cord clamping group, and infants in the early cord clamping group were more likely to be iron-deficient at 3 to 6 months (RR 2.65, 95% CI 1.04 to 6.73).

In preterm infants, a 2012 meta-analysis of 15 studies (738 infants born at between 24 and 36 weeks’ gestation) found that delaying cord clamping for 30 to 180 seconds was associated with less IVH (RR 0.59, 95% CI 0.41 to 0.85), decreased need for transfusions for anemia (RR 0.61, 95% CI 0.46 to 0.81), and lower risk for necrotizing enterocolitis compared with immediate clamping ^34^. However, there were no clear differences in severe (grade 3 or 4) IVH and periventricular leukomalacia. A later trial comparing immediate with delayed cord clamping for 30 seconds among preterm neonates born at between 24 and 34 weeks of gestation found a lower rate of IVH among neonates in the delayed cord clamp group compared with neonates in the immediate clamp group but this was not statistically significant ^35^. A trial assessing the effects of delayed cord clamping in 208 preterm (<32 weeks’ gestation) infants on neonatal and 18-month motor outcomes found that although delayed cord clamping did not alter the incidence of IVH in preterm infants, it improved motor function at 18 to 22 months’ corrected age (odds ratio 0.32, 95% CI 0.10 to 0.90) ^36^. More recently, a meta-analysis of 18 RCTs comparing delayed versus early clamping in 2834 infants born at less than 37 weeks’ gestation found that delayed clamping (30 seconds to more than 120 seconds) reduced hospital mortality (RR 0.68, 95% CI 0.52 to 0.90); however, delayed cord clamping did not reduce the incidence of intubation for resuscitation, mechanical ventilation, IVH, or brain injury ^37^. Maternal postpartum hemorrhage or the need for maternal blood transfusion was not impacted by delayed clamping.

As a result, the American College of Obstetricians and Gynecologists recommends a delay in umbilical cord clamping for at least 30 to 60 seconds after birth in vigorous term and preterm infants ^38^. This has been endorsed by the American Academy of Pediatrics, and recent Neonatal Resuscitation Program guidelines recommend delayed umbilical cord clamping for at least 30 to 60 seconds for most vigorous term and preterm infants ^39, 40^.

It has been postulated that delayed cord clamping allows improved cardiovascular transition with resultant improved cerebral autoregulation ^41^. Also, delaying clamping for at least 60 seconds may increase the number of infants breathing before the cord is clamped and this may decrease need for invasive mechanical ventilation and endotracheal intubation ^37^. Animal data suggest that timing cord clamping on the basis of the infant’s physiology may optimize the potential benefits and that delayed cord clamping may be of greatest benefit to apneic infants ^42– 44^.

## Non-steroidal anti-inflammatory drugs

Indomethacin, a non-selective cyclo-oxygenase (COX) inhibitor was shown to reduce the incidence of IVH in preterm infants (RR 0.66, 95% CI 0.53 to 0.82) ^45^. A meta-analyses of 19 large RCTs found that prophylactic indomethacin in preterm infants did not improve mortality or long-term developmental outcomes ^46^. However, pooled data from recent observational studies suggest that the use of prophylactic indomethacin may be associated with a small reduction in mortality risk, particularly in infants with birth weights above the 10th percentile ^47^.

Ibuprofen is another non-selective COX inhibitor but has not been shown to prevent IVH in premature infants ^48^.

Indomethacin promotes maturation of the cerebral vasculature ^49^; blunts cerebral vascular responses caused by hypoxia, hypercapnia, hypertension, and asphyxia ^50, 51^; and improves cerebral vascular autoregulation ^52^, all of which may contribute to a reduction of IVH.

Prophylactic indomethacin administration continues to be used in many centers across the United States despite conflicting evidence. Well-designed contemporary studies are required to guide clinical practice.

## Therapeutic hypothermia

Multiple RCTs of therapeutic hypothermia in term newborns have demonstrated that hypothermia (33–35 °C) for 72 hours starting within about 6 hours of birth is associated with improved survival and decreased neurological impairment ^53– 59^. A meta-analysis ^60^ of 11 of these trials involving 1505 term and late preterm infants with moderate or severe encephalopathy found that therapeutic hypothermia resulted in decreased death or major disability by 18 to 24 months of age (RR 0.75, 95% CI 0.68 to 0.83), as well as decreased mortality (RR 0.75, 95% CI 0.64 to 0.88), and reduced neurodevelopmental disability in survivors (RR 0.77, 95% CI 0.63 to 0.94). Subgroup analysis revealed that infants with severe encephalopathy demonstrated significant reduction in mortality but no significant reduction in major disability, although there was a trend toward improvement (RR 0.75, 95% CI 0.50 to 1.12), and the lack of significance was attributed to the small number of infants in this category. There was no significant reduction in death or moderate to severe disability at 6 to 7 years of age among those that underwent hypothermia, but there was a clinically important trend toward improvement (RR 0.81, 95% CI 0.64 to 1.04) and a significant reduction in death at 6 to 7 years of age. The CoolCap trial, for instance, found that the measured outcome at 18 months was strongly associated with overall functional scores at 7 to 8 years of age, supporting a sustained treatment effect of therapeutic hypothermia ^61^. The NICHD (Eunice Kennedy Shriver National Institute of Child Health and Human Development) trial found no significant reduction in the combined outcome of death or an IQ score of less than 70 at 6 to 7 years in the hypothermia group; however, hypothermia resulted in lower death rates and did not increase rates of severe disability among survivors ^62^.

The above-mentioned meta-analysis also demonstrated a significant reduction in CP in the hypothermia groups (RR 0.66, 95% CI 0.54 to 0.82) ^60^. Therapeutic hypothermia was also associated with significant reduction in the presence of abnormal findings on MRI ^60^, in particular in the basal ganglia or thalamus, white matter, and abnormal posterior limb of the internal capsule ^63^. A retrospective cohort study of 224 neonates found that therapeutic hypothermia in moderate encephalopathy was associated with reduced seizures (RR 0.43, 95% CI 0.30 to 0.61) ^64^.

It remains to be seen whether the therapeutic window for hypothermia may extend beyond 6 hours. A multicenter RCT spanning 8 years and including term infants with moderate or severe HIE found that hypothermia initiated at 6 to 24 hours after birth compared with non-cooling resulted in a 76% probability of any reduction in death or disability at 18 to 22 months ^65^. The neuroprotective mechanisms of hypothermia include reduced concentrations of free creatine, lactate, NAA, and neurotransmitters such as glutamate, glutamine, GABA, and aspartate and increased concentration of taurine and phosphocreatine. Animal models have also demonstrated that hypothermia reduces synthesis of free radicals and nitric oxide and suppression of microglial activation ^66^. Overall, hypothermia attenuates cellular energy demand and secondary energy failure ^67^.

Although therapeutic hypothermia is now the standard of care for term and late preterm infants with moderate/severe HIE, future directions include investigating the neuroprotective mechanism in infants with mild encephalopathy and in preterm infants. There is recent evidence to suggest that mild HIE is associated with disability ^68^. In addition, the combination of hypothermia with other therapeutic agents such as those described below is being investigated.

## Recombinant human erythropoietin

Several studies suggest that erythropoietin, either alone or in combination with hypothermia therapy, improves neurodevelopmental outcomes and is safe. A case control study in Egypt with 45 neonates with mild to moderate HIE found that neonates that received human recombinant erythropoietin 2500 IU/kg subcutaneously daily for 5 days had decreased serum nitrous oxide concentrations, fewer seizures, improved electroencephalogram backgrounds, and favorable neurologic outcomes at 6 months of age. An RCT in China in 167 term neonates with moderate to severe hypoxia-ischemia demonstrated that erythropoietin monotherapy 300 to 500 IU/kg reduced disability at 18 months in infants with moderate but not severe injury ^69^. A trial in India in 100 term neonates with moderate or severe HIE found that erythropoietin 500 U/kg monotherapy given within 6 hours of birth resulted in significant reduction of death or moderate or severe disability at 19 months of age (RR 0.57, 95% CI 0.38 to 0.85) and lower risk of CP in survivors (RR 0.52, 95% CI 0.25 to 1.03). A phase II, multicenter, double-blinded controlled trial in the Unites States (NEATO) in term newborns with moderate to severe HIE found that multiple doses of erythropoietin (1000 U/kg) given intravenously for 7 days was associated with reduced severity of brain injury on neonatal MRI, specifically in the subcortical region, and improved motor function at 1 year among infants undergoing therapeutic hypothermia ^70^. Phase III trials are under way to determine whether high-dose erythropoietin in conjunction with hypothermia in infants with moderate/severe HIE reduces the combined outcome of death or neurodevelopmental disability and improves neurodevelopmental outcomes at 2 years of age, without significant adverse effects, when compared with hypothermia alone ^71^. A pilot prospective study of nine patients who met criteria for hypothermia suggests that combination therapy with 300 U/kg erythropoietin every other day for 2 weeks, 250 mg/kg magnesium sulfate for 3 days, and therapeutic hypothermia is feasible in newborns with HIE. Phase II and II studies are needed to investigate the neuroprotective effect of this strategy.

However, it should be noted that a recent mouse model study suggested that, when used immediately after the insult, erythropoietin may not be beneficial in situations of extreme oxidative stress and may, in fact, worsen the injury ^72^.

Preliminary data also suggest a benefit of erythropoietin in preterm infants. A retrospective analysis ^73^ of neurodevelopmental outcome data from extremely-low-birth-weight infants given 500 to 2500 U/kg erythropoietin × 3 doses in a phase I/II trial ^74^ found that erythropoietin administration correlated with improvement of cognitive and motor scores. A study of 102 infants reported improved cognitive scores at 18 to 22 months in preterm infants that received low doses of erythropoietin (400 U/kg, 3×/week subcutaneously) or darbepoetin (10 μg/kg, 1×/week subcutaneously) ^75^. In a large multicenter placebo-controlled randomized trial in Switzerland of very preterm infants (born at between 26 and 32 weeks), there were no significant differences in neurodevelopmental outcomes at 2 years between those that received prophylactic early high-dose erythropoietin for neuroprotection and those that received placebo ^76^. However, subgroup analyses revealed that high-dose erythropoietin administration was associated with reduced brain injury, improved white matter development in the major white matter tracts, and an increase of local structural connectivity strengths ^77– 79^. A large RCT of 800 infants of not more than 32 weeks’ gestational age demonstrated that repeated low-dose erythropoietin (500 IU/kg) reduced risk of long-term neurological disability in very preterm infants at 18 months of age (RR 0.40, 95% CI 0.27 to 0.59) ^80^. A meta analyses of four RCTs including 1133 preterm infants showed that prophylactic erythropoietin improved neurocognition at 18 to 24 months’ corrected age but had no significant effect on motor development, hearing, or vision ^81^.

A recent Cochrane review of 34 studies spanning 22 countries enrolling 3643 infants, gestational age of less than 37 weeks and/or birth weight of less than 2500 g concluded that early treatment with erythropoiesis-stimulating agents significantly decreased rates of IVH, periventricular leukomalacia, and necrotizing enterocolitis ^82^. It also found a reduction in any neurodevelopmental impairment at 18 to 22 months in the erythropoietin group compared with the placebo group (typical RR 0.62, 95% CI 0.48 to 0.80), but the quality of evidence was deemed to be low.

Further trials are needed to determine optimal dosing strategy and long-term assessment of developmental outcomes. The Phase 3 Preterm Erythropoietin Neuroprotection (PENUT) trial (ClinicalTrials.gov Identifier: NCT01378273) randomly assigned 941 preterm infants between 24 and 27 weeks’ gestation to receive erythropoietin 1000 U/kg or placebo given intravenously every 48 hours for six doses, followed by 400 U/kg or sham injections three times a week through 32 weeks postmenstrual age ^83^. Results are pending publication. Other trials using erythropoietin in preterm or very preterm infants (ClinicalTrials.gov Identifiers: NCT02550054 and NCT02076373) are under way to assess neurodevelopmental outcomes ^84, 85^.

The neuroprotective and neuroregenerative effects of erythropoietin are likely related to its anti-inflammatory ^86^, anti-excitotoxic, anti-oxidant ^87^, and anti-apoptotic effects on neurons and oligodendrocytes and regenerative effects of oligodendrogenesis, neurogenesis, and angiogenesis ^88– 92^.

## Melatonin

Data from animal studies suggest a role of melatonin in perinatal neuroprotection ^93– 97^. In a randomized controlled pilot study of 45 newborns, 30 of whom had HIE, melatonin administration together with hypothermia was associated with fewer seizures, fewer white matter abnormalities on MRI, and better mortality rate at 6 months without developmental or neurological abnormalities ^98^. A phase II multi-center double-blinded randomized placebo-controlled trial (Mint study) evaluating the neuroprotective effect of intravenous melatonin in 58 preterm infants born at less than 31 weeks’ gestation found no difference in white matter fractional anisotropy ^99^. The PREMELIP study aimed to assess the neuroprotective effect of melatonin administered in the immediate prepartum period in very preterm infants (<28 weeks’ gestation) using MRI but was terminated ^100^. The “Protect Me Trial”, which aims to evaluate the effect of maternal melatonin supplementation in pregnancies with early-onset fetal growth restriction on neurodevelopmental outcomes at 2 years of age, is under way ^101^.

Melatonin’s neuroprotective effects are likely due to its antioxidant ^102, 103^, anti-inflammatory ^94, 96, 104^, and anti-apoptotic ^94, 105^ effects, which may protect against free radical–induced damage incurred during times of increased oxidative stress perinatally ^106^.

## Xenon

Xenon has demonstrated neuroprotection in animal models of moderate HIE and this effect is enhanced when combined with cooling ^107, 108^. However, a single phase II trial randomly assigning 92 newborns with moderate to severe HIE to either cooling plus xenon or cooling alone did not show significant differences between magnetic resonance biomarkers of brain damage or in occurrence of seizures during primary hospitalization ^56^. Long-term neurodevelopmental outcomes were not reported. However, this study was limited by delay before starting xenon (median of 11 hours). Thus, current evidence is inadequate to determine whether xenon therapy for newborns with HIE is effective ^109^.

Xenon’s neuroprotective effects are thought to be related to its inhibition of NMDA subtype of the glutamate receptor, a key step in the neurotoxic cascade, and activation of two species of potassium channels which have been linked to neuroprotection ^110^.

## Topiramate

Topiramate has demonstrated neuroprotective effects in animal models of transient global cerebral ischemia, ischemic stroke, and neonatal hypoxic ischemic cerebral injury ^111– 113^. A phase II trial in term newborns with moderate to severe HIE treated with hypothermia showed that treatment with topiramate was safe but that, compared with cooling alone, it did not improve death or neurological disability ^114^. There was a reduction in the prevalence of epilepsy observed in the topiramate group. The neuroprotective properties of topiramate are presumed to be due to AMPA and kainate receptors inhibition ^115^, blockade of sodium ^116^ and high voltage-activated calcium currents, and inhibitory effect on mitochondrial permeability transition pores ^117, 118^.

## Allopurinol

A 2012 Cochrane review including 114 infants in three trials found no clear differences in severe neurodevelopmental disability or death among survivors at 18 months or at 4 to 8 years after allopurinol versus placebo (RR 0.78, 95% CI 0.56 to 1.08) ^119^. In addition, a follow-up study of two of the trials included in the above review found no differences in mortality or developmental disability at the age of 4 to 8 years in the overall group of asphyxiated infants; however, a subgroup revealed significantly less severe adverse outcome in the allopurinol-treated moderately asphyxiated infants compared with controls (RR 0.40, 95% CI 0.17 to 0.94) ^120, 121^. A more recent follow-up study of 222 women in labor with suspected fetal hypoxia randomly assigned to receive allopurinol or placebo demonstrated that allopurinol administration does not improve long-term developmental and behavioral outcome at 5 years of age ^121, 122^. Currently, a multicenter European trial (ClinicalTrials.gov Identifier: NCT03162653) is under way to evaluate whether early postnatal allopurinol in addition to standard of care reduces the incidence of death or severe neurodevelopmental impairment at 24 months of age in newborns with HIE ^123^.

Allopurinol, a xanthine oxidase inhibitor, preserves NMDA receptor integrity and prevents adenosine degradation and oxygen radical formation and this potentially confers neuroprotection in HIE ^124^.

## Autologous cord blood cell therapy

Preclinical evidence is emerging to support the use of cord-derived mesenchymal stromal cells (MSCs) for regeneration and repair of injured immature brain ^125, 126^. Animal models suggest that exogenous administration of MSCs significantly reduces brain injury and post-hemorrhagic hydrocephalus after IVH by protecting against inflammation, gliosis, and apoptosis of the injured brain ^127– 129^.

Limited clinical data exist suggesting that the use of autologous cord blood cells for perinatal/preterm brain injury is safe and feasible ^130– 132^. Further clinical trials are under way to evaluate safety and efficacy of autologous cord blood cells for neonatal brain injury ^133– 138^.

MSCs are thought to restore neurological injury by differentiation to neuronal cells or, more importantly, via secretion of paracrine factors such as insulin-like growth factor (IGF-1), vascular endothelial growth factor (VEGF), and brain-derived neurotrophic factor (BDNF), which augment neuronal and glial cell proliferation and survival ^139, 140^. These transplanted MSCs secrete the paracrine factors at variable levels in response to cues from the local substrate ^141^. Moreover, MSCs are shown to secrete anti-inflammatory cytokines ^127^.

## Vitamin E

A meta-analysis of 26 randomized clinical trials found that vitamin E supplementation in preterm infants (gestational age less than 37 weeks or birth weight less than 2500 g) reduced the risk of intracranial hemorrhage but increased the risk of sepsis ^142^. Currently, there are no data to support the use of vitamin E for perinatal neuroprotection.

## Creatine

Animal experiments demonstrate that, when given as a supplement to the mother’s diet during pregnancy, creatine protects the fetal brain against hypoxic insult at term ^143– 145^. Further trials are needed to evaluate the effect of antenatal creatine supplementation on neuroprotection of the fetus. 

Creatine is involved with cellular energy production but also has demonstrated antioxidant actions ^146^, stabilization of lipid membranes ^147^, and interactions with glutamate and GABAA receptors ^148^ that diminish excitotoxicity ^145, 149^.

## Conclusions

Recent clinical and laboratory advances in neuroprotection of the developing brain suggest that there is a cascade of biochemical events that can be partially disrupted, leading to reduced brain injury. Brain cooling and blockade of NMDA glutamate receptors are two of the earliest interventions that showed an ability to reduce brain injury and these interventions can be synergistic. Cooling has been shown to reduce brain injury in human term infants by impeding the cascade of injury, especially the events in the mitochondria. Magnesium has shown neuroprotective activity in numerous studies, several possibly by anti-inflammatory and anti-glutamate effects. Anti-erythropoietin protective effects have also been identified. Recent advances in perinatal neuroprotection are growing briskly as we identify more potential therapeutic targets.
